# Field evaluation of SD BIOLINE HIV/Syphilis Duo assay among pregnant women attending routine antenatal care in Juba, South Sudan

**DOI:** 10.1371/journal.pone.0205383

**Published:** 2018-10-10

**Authors:** Dennis K. Lodiongo, Bior K. Bior, Gregory W. Dumo, Joel S. Katoro, Juma J. H. Mogga, Michael L. Lokore, Abe G. Abias, Jane Y. Carter, Lul L. Deng

**Affiliations:** 1 Clinical Research and Molecular Diagnostics Unit, National Public Health Laboratory, Ministry of Health, Juba, Republic of South Sudan; 2 Parasitology Unit, National Public Health Laboratory, Ministry of Health, Juba, Republic of South Sudan; 3 Centers for Disease Control and Prevention, Juba, Republic of South Sudan; 4 National Tuberculosis Programme, Ministry of Health, Juba, Republic of South Sudan; 5 Bacteriology Unit, National Public Health Laboratory, Ministry of Health, Juba, Republic of South Sudan; 6 Quality Assurance Unit, National Public Health Laboratory, Ministry of Health, Juba, Republic of South Sudan; 7 Amref International University, Nairobi, Kenya; 8 Administration and Management, National Public Health Laboratory, Ministry of Health, Juba, Republic of South Sudan; Epicentre, CAMEROON

## Abstract

The SD BIOLINE HIV/Syphilis Duo assay is the first World Health Organization prequalified dual rapid diagnostic test for simultaneous detection of HIV and *Treponema pallidum* antibodies in human blood. Prior to introducing the test into antenatal clinics across South Sudan, a field evaluation of its clinical performance in diagnosing both HIV and syphilis in pregnant women was conducted. SD Bioline test performance on venous blood samples was compared with (i) Vironostika HIV1/2 Uniform II Ag/Ab reference standard and Alere Determine HIV 1/2 non-reference standard for HIV diagnosis, and (ii) *Treponema pallidum* hemagglutination reference standard and Rapid plasma *reagin* non-reference standard for syphilis. Sensitivity, specificity, positive predictive value (PPN), negative predictive value (NPV) and kappa (κ) value were calculated for each component against the reference standards within 95% confidence intervals (CIs); agreements between Determine HIV 1/2 and SD Bioline HIV tests were also calculated. Of 442 pregnant women recruited, eight (1.8%) were HIV positive, 22 (5.0%) had evidence of syphilis exposure; 14 (3.2%) had active infection. For HIV diagnosis, the sensitivity, specificity, PPV and NPV were 100% (95% CI: 63.1–100), 100% (95% CI: 99.2–100), 100% (95% CI: 63.1–100) and 100% (95% CI: 99.2–100) respectively with κ value of 1 (95% CI: 0.992–1.000). Overall agreement of the Duo HIV component and Determine test was 99.1% (95% CI: 0.977–0.998) with 66.7% (95% CI: 34.9–90.1) positive and 100% (95% CI: 0.992–1.000) negative percent agreements. For syphilis, the Duo assay sensitivity was 86.4% (95% CI: 65.1–97.1) and specificity 100% (95% CI: 99.1–100) with PPV 100% (95% CI: 82.4–100), NPV 99.2% (95% CI: 97.9–99.9) and κ value 0.92 (95% CI: 0.980–0.999). Our findings suggest the SD Bioline HIV/Syphilis Duo Assay could be suitable for HIV and syphilis testing in women attending antenatal services across South Sudan. Women with positive syphilis results should receive treatment immediately, whereas HIV positive women should undergo confirmatory testing following national HIV testing guidelines.

## Background

Globally, an estimated one million syphilis infections [1] and 1.5 million human immunodeficiency virus (HIV) infections [2] occur in pregnant women annually. Approximately 30% of pregnant women with untreated syphilis will have stillbirths or neonatal death, and another 30% will deliver babies with congenital syphilis, a condition with a mortality of up to 50% [3]. Syphilis infection during pregnancy also increases the risk of mother-to-child transmission (MTCT) of HIV [4]. Syphilis infection in adults causes epithelial and mucosal breaches facilitating sexual transmission of HIV virions. In addition, *Treponema pallidum* (*T*. *pallidum*) and its pro-inflammatory components induce expression of chemokine receptor type 5 (CCR5) on human monocytes, thereby enhancing the susceptibility of these cells to HIV infection [5]. Early screening and effective treatment of syphilis cures syphilis in pregnancy and prevents congenital syphilis and neonatal deaths [6]. Integrating rapid syphilis testing into national HIV programmes in South Sudan would prevent an estimated 1,125 congenital syphilis cases and 1,223 stillbirths and neonatal deaths annually, with a potential cost saving of 525,000 USD [7]. Timely HIV diagnosis in pregnant women is crucial for effective prevention of MTCT and optimal clinical management of HIV [2]. Screening for HIV and syphilis in pregnant women is recommended by the World Health Organization (WHO) to reduce the morbidity and mortality associated with these undiagnosed and untreated infections [8].

In South Sudan, syphilis and HIV continue to be major public health concerns. Sentinel surveillance at antenatal clinics has estimated the prevalence of syphilis and HIV at 8.3% and 2.6% respectively in pregnant women [9]. In 2011, the country introduced concurrent HIV and syphilis screening of pregnant women using separate HIV and *T*. *pallidum* rapid diagnostic tests (RDT) but often pregnant women are not screened for syphilis due to shortage of test kits. Recently, the SD BIOLINE HIV/Syphilis Duo RDT (Standard Diagnostics, Inc. Yongin, Gyeonggi, South Korea) was developed for simultaneous detection of HIV and syphilis infections in human serum, plasma or whole venous or capillary blood. The Duo RDT is designed to qualitatively detect IgG, IgM and IgA antibodies to HIV-specific antigens (HIV-1 gp41, sub O, HIV-2 gp36) and specific IgM and IgG antibodies to a 17 kilodalton recombinant *T*. *pallidum* antigen (*rTp*17 kDa). This assay is user friendly, has a turnaround time of about 25 minutes and costs approximately 2 USD per test, compared to current techniques that cost 2 USD for TPHA and 3–10 USD to complete the HIV algorithm [10]. Although the SD BIOLINE HIV/syphilis Duo RDT is WHO prequalified [11], its diagnostic usefulness has not been evaluated in South Sudan. To adopt this assay for routine use in antenatal clinics across the country, a field evaluation of its clinical performance in diagnosing both HIV and syphilis in pregnant women was undertaken.

## Methods and materials

### Study design, sites and population

This field-based cross-sectional study was conducted at Juba Teaching Hospital, and Munuki, Gurei and Nyakuron Primary Health Care Centres in Juba, South Sudan. At each facility, midwives identified eligible participants among pregnant women attending routine antenatal care (ANC) services and recorded their characteristics in ANC cards and registers.

### Inclusion criteria

Pregnant women aged 15–49 years presenting for their first ANC visit and who consented to participate in the study.

### Exclusion criteria

Participants presenting for second or further ANC visits and those who refused to provide informed written consent.

### Sampling method and sample size

A convenience sampling technique was used to recruit 442 participants into the study over a period of three months.

### Blood collection and testing at ANC sites

Using an aseptic technique, 5 mL of venous blood was collected from each participating pregnant woman into a pre-labelled plain vacutainer tube by pre-trained midwives. Midwives performed and interpreted the SD BIOLINE HIV/Syphilis Duo assay according to the manufacturer’s instructions (Standard Diagnostics, Inc. Yongin, Gyeonggi, South Korea). Results were recorded in worksheets, counter-checked by a technician and sealed in envelopes. The remaining blood specimens were placed in a cool box and transported together with results to the Public Health Laboratory (PHL) in Juba for further testing and data analysis.

### Laboratory testing at Public Health Laboratory (PHL)

Blood specimens were centrifuged (Thermo SCIENTIFIC, CL10 Centrifuge) at 1000 rpm for 5 minutes and serum transferred into 1.5 mL pre-labelled Eppendorf tubes. If testing was not done on the same day of specimen reception, sera were kept at 2–8°C for not more than 24 hours before analysis. Each serum specimen was tested for syphilis using the Rapid plasma *reagin* (RPR) test (Fortress Diagnostics Limited, Antrim, UK) as a non-reference standard and a reference standard *Treponema pallidum* hemagglutination (TPHA) assay (BIOTEC Lab21 Healthcare Ltd, Dorset, UK) by two independent technicians blinded to each other’s results and the results of the Duo assay. RPR reactive samples were titrated in two-fold serial dilutions to obtain endpoint titre values. For HIV reference testing, all specimens were tested with Vironostika HIV1/2 Uniform II Ag/Ab assay (Biomérieux SA, Marcy-I Etoile, France) and positive specimens retested in duplicate wells. The specimens were also tested for HIV using a non-reference standard, the Alere Determine HIV1/2 assay (Alere Medical Co. Ltd., Matsuhidai, Matsudo-shi, Chiba, Japan). Reactive specimens were confirmed with the Uni-Gold Recombinant HIV1/2 assay (Trinity Biotech, Bray, Ireland). All test procedures were conducted following the manufacturer’s instructions for each test.

For both the Determine/Uni-Gold HIV1/2 combination and Vironostika HIV1/2 Uniform II Ag/Ab tests, testing was conducted by independent laboratory technicians blinded to each other’s results and the results of the Duo assay.

### Data analysis

All test results were entered into an Excel spreadsheet and reviewed for accuracy prior to data analysis. Sensitivity, specificity, Cohen’s kappa coefficient (κ value), positive predictive value and negative predictive value of the Duo kit were calculated using OpenEpi, Version 3.01. To compare results of the Duo HIV test component with results of the Determine HIV 1/2 screening test of the national HIV test algorithm, overall test agreement was calculated. The samples for which there was agreement for both positive and negative test results were summed and divided by the total number of samples tested. To differentiate agreements between positive and negative results, the positive percent agreement and the negative percent agreement were calculated using a 2 by 2 table. This method was chosen because it allows comparison of tests in which neither is a reference standard [12, 13]. The 95% confidence intervals (CIs) for each diagnostic parameter were calculated using an exact binomial method.

### Ethical considerations

Study approval was granted by the Research and Ethics Committee of the Ministry of Health, Republic of South Sudan. The Committee considered and approved the study as no risk to foetuses. Recruitment into the study was voluntary and each enrolled participant provided informed written consent. To maintain confidentiality, study data were protected by removing personal identifiers. Unique identification numbers assigned to each participant by the midwives accompanied the specimens and results for reference testing and analysis at the PHL.

## Results

Four hundred and forty two (442) venous blood specimens were collected from pregnant women, and tested and analysed between June and August 2016. Eight (1.8%) of 442 pregnant women were HIV positive, 22 (5.0%) had evidence of syphilis exposure while 14 (3.2%) of these were active infections. These results together with the sensitivity and specificity of the SD Bioline Duo test are shown in Table 1.

**Table 1:** Diagnostic accuracy of the SD Bioline Duo HIV/syphilis assay among pregnant women attending ANC in Juba, South Sudan.

Blood Specimens tested	Reference standards	Number of positive specimens (n)	Number of negative specimens (n)	Sensitivity % (95% CI)	Specificity % (95% CI)	PPV % (95% CI)	NPV %(95% CI)	κappa value (95% CI)
HIV N = 442	Vironostika HIV1/2 Uniform II Ag/Ab test	8	434	100 (63.1–100)	100 (99.2–100)	100 (63.1–100)	100 (99.2–100)	1 (0.992–1.000)
Syphilis N = 442	TPHA Test*	22	420	86.4 (65.1–97.1)	100 (99.1–100)	100 (82.4–100)	99.3 (97.9–99.9)	0.92 (0.980–0.999)

*Treponemal pallidum hemagluttination assay (TPHA) (Fortress Diagnostics Limited, Antrim, UK)

CI: Confidence Intervals, PPV: Positive Predictive value, NPV: Negative Predictive Value

The SD Bioline Duo test resulted in three false negative syphilis results which were RPR reactive with titre values between 1 in 4 and 1 in 16. Fourteen of the 22 TPHA positive specimens were RPR reactive suggesting active syphilis infection while eight were RPR non-reactive implying previous infection. The titre values for all 14 RPR reactive specimens ranged from 1 in 2 to 1 in 32. The SD Bioline Duo test only detected *T*. *pallidum* antibodies in 11 of the 14 RPR reactive specimens and in all the eight RPR non-reactive specimens.

The Determine HIV 1/2 screening test detected 12 reactive specimens while eight of these specimens tested HIV positive on the Duo test as shown in Table 2. Overall agreement between the Duo HIV test results and the Determine HIV 1/2 test results was 99.1% (438/442) (95% CI: 97.7–99.8).

**Table 2:** Comparison of the diagnostic agreement between SD Bioline Duo HIV test component results and the Alere Determine^TM^ HIV 1/2 test results.

		Non-reference standard*		No. of samples (n)	Postive agreement (95% CI)	Negative agreement (95% CI)
		Positive	Negative			
**Duo test**	Positive	8	0	8	66.67% (34.9–90.1)	100% (99.2–100)
	Negative	4^¥^	430	434		
Total (n)		12	430	**442**		

*Alere Determine HIV 1/2 test, the screening test for South Sudan National HIV test algorithm.

¥Tested negative on Uni-Gold HIV1/2 test, the confirmatory test for the National HIV test algorithm and negative on the HIV reference standard.

## Discussion

The present data showed overall good clinical performance of the SD BIOLINE HIV/Syphilis Duo RDT for detection of both HIV and syphilis antibodies in pregnant women attending routine antenatal services in Juba, South Sudan. Eight participants were confirmed HIV positive while 22 had evidence of syphilis antibodies with 14 active infections. No participant was co-infected. All HIV and syphilis confirmed cases were managed by each facility following standard protocols.

The HIV component of the Duo RDT had a sensitivity of 100% (95% CI: 63.1 to 100) and specificity of 100% (95% CI: 99.2 to 100). The concordance of the HIV component of the Duo RDT with HIV 1/2 Uniform II Ag/Ab reference assay was almost perfect (kappa 1.0 (95% CI: 0.992 to 1.000)). These findings are consistent with the results of similar studies [10, 14–17]. Globally, HIV RDTs, including some with lower field evaluation sensitivity and specificity compared with laboratory evaluations, have remained the mainstay of HIV diagnosis [18, 19]. WHO recommends at least 99% sensitivity and 98% specificity for HIV antibody RDTs [20]; the Duo RDT HIV component met this requirement in our field evaluation and therefore would be a good alternative screening test for HIV antibodies.

When the results of the SD Bioline Duo HIV test component were compared with the national HIV test algorithm’s screening test (Alere Determine), the Determine detected 12 reactive specimens while the Duo test detected 8 HIV positive specimens. This resulted in 99.1% (95% CI: 97.7–99.8) overall agreement with 66.7% (95% CI: 34.9 to 90.1) positive percent agreement and 100% (95% CI: 99.2 to 100) negative percent agreement. The positive percent agreement differed from that of Olugbenga *et al*.[21] but agreed with the negative percent agreement probably due to the four discordant results in this study. Besides, Olugbenga *et al*. compared the performance of the duo test HIV component with the national HIV test algorithm unlike this study. Four of the specimens that formed part of the agreement data analysis (Table 2) tested reactive with HIV Determine but tested negative with Uni-Gold, the confirmatory test in the South Sudan national HIV test algorithm. However, these four specimens also tested negative with the Vironostika HIV1/2 Uniform II Ag/Ab reference standard and were thus interpreted as negative specimens (Table 1). Therefore, in addition to the low false positive rate of the Duo test HIV component, taken together, these results suggest the Duo test could play a vital role in the South Sudan National HIV test algorithm.

In South Sudan, the *T*. *pallidum* Duo RDT component showed good sensitivity at 86.4% (95% CI: 65.1 to 97.1) and 100% (95% CI: 99.1 to 100) specificity with almost perfect concordance (kappa 0.92 (95% CI: 0.980 to 1.000)). These results are comparable to results of similar studies from other countries (Table 3) [8, 10, 11, 17, 22].

**Table 3:** SD Bioline Duo test syphilis results for field studies verses laboratory-based evaluations.

Study	Country	Specimen type	Sensitivity (95% CI)	Specificity (95% CI)
Current Study	Juba, South Sudan	Venous whole blood	86.4% (66.7–95.3)	100% (99.1–100)
Bristow *et al*, 2016	Lima, Peru	Fingerprick whole blood	89.2% (83.5–93.5)	98.8% (96.5–99.8)
Black *et al*, 2016	Johannesburg, South Africa	Fingerprick whole blood	66.8% (52.0–78.9)	99.5% (94.5–99.3)
WHO report, 2015	Geneva, Switzerland	Serum/Plasma	87% (81.5–91.3)	99.5% (97.2–100)
Bristow *et al*, 2016	Port-au-Prince, Haiti	Fingerprick whole blood	100% (81.5–100)	96.8% (83.3–99.9)
Omoding *et al*, 2014	Southwestern Uganda	Plasma	100% (79.0–100)	100% (97.6–100.0)

Differences in results could be explained by specimen type, study design, reference method or disease stage as reported for syphilis single RDTs [23–27]. Following syphilis infection, early immune responses are directed against TpN47, followed by TpN15 and TpN17 antigens [28], so this could further explain the low sensitivity of the Duo RDT since it contains recombinant Tp17 antigen only.

If we had used the syphilis test algorithm of RPR screening followed by TPHA confirmation of reactive specimens, eight specimens would have tested false positive with the Duo assay. This approach could have decreased the Duo kit sensitivity to 78.6% (11/14) and specificity to 98.1% (420/428). The International Diagnostics Centre recommends the target product profile (TPP) for an ideal dual HIV/syphilis RDT, *treponemal* test component to have a minimum sensitivity of 75% and specificity of 90% [29] which agree with the results from this study. In routine syphilis diagnosis, the SD Bioline Duo RDT results would require confirmatory RPR testing before treatment initiation. However, for screening for the presence of *treponemal* antibodies in pregnant women, RPR confirmation for active infection may not be necessary since all pregnant women with a positive *treponemal* test are treated immediately because the risks associated with untreated syphilis outweigh the minimal costs and risk of over-treatment of mothers with a false positive result [30]. Studies in prenatal populations have documented that RPR titres ≥ 1in 8 could be important for pregnant women as this has been significantly associated with adverse pregnancy outcomes [31].

Three out of the 14 RPR reactive and TPHA positive specimens tested negative with the Duo test syphilis component. Of these three, one had a low RPR titre of 1 in 4 while the other two had higher titres of 1 in 16. We could not explain these results which differed from Black *et al*. [17] who found specimens with high RPR titres (≥ 1 in 8) are likely to test positive for *T*. *pallidum* antibodies with the Duo test. On the other hand, taking a titre of ≥ 1 in 8, the Duo test would have had 77.8% (7/9) sensitivity but 80% (4/5) sensitivity if a titre of ≤1 in 4 was considered, excluding the eight RPR non-reactive but TPHA positive specimens. These results are not dissimilar to those of Causer *et al*. who found sensitivity of 94.6% with a titre of ≥ 1 in 8 and 76.3% for a titre of ≤1 in 4 for a single Bioline syphilis RDT [24]. Due to the inconsistency in RPR values compared with the Duo test’s ability to detect antibodies, we think it would be appropriate to compare TPHA but not RPR results with the performance of the Duo test, as shown by Zorzi *et al*. This study documented that for *Treponema pallidum* particulate agglutination (TPPA) test titres >1 in 1280, the misclassification rate for the syphilis *treponemal* tests would be very low [32]. This finding is consistent with the *treponemal* antigen-based syphilis testing component of the Duo test. In this study, we did not determine TPHA titres making comparison of our results with Zorzi *et al*. not possible. Given the paucity of studies comparing the performance of syphilis *treponemal* RDTs based on different TPHA/TPPA titre cut off values, we suggest evaluation studies be undertaken to assess the potential impact of different TPHA/TPPA titre cut-off values on the performance of the SD Bioline Duo *treponemal* test component or any RDT *treponemal* test as opposed to comparing it to RPR titre values.

Study limitations include: Using venous blood specimens drawn from participants for multiple analyses such as ABO Rh grouping and haemoglobin estimation instead of capillary blood considering field use. However, since published data in the literature show good diagnostic accuracy with capillary blood as recommended by the manufacturer, we would not hesitate to advise our facilities to use capillary blood when needed. Secondly, although the SD Bioline Duo kit is promising in terms of diagnostic accuracy, the sensitivities and positive predictive values, although not dissimilar from those reported in the literature, may be imprecise due to the small number of positive samples for HIV and syphilis infections tested in this study.

## Conclusion

The SD BIOLINE HIV/syphilis Duo RDT showed good clinical field performance with venous blood specimens from pregnant women attending antenatal clinics in Juba, South Sudan; having almost perfect concordance with the reference standards. The Duo RDT performed better for HIV than for syphilis diagnosis. It also performed better than the Determine HIV 1/2 screening test in terms of ruling out an HIV infection. This RDT kit offers an opportunity for simultaneous screening of both HIV and syphilis in pregnant women across our antenatal care and prevention of mother to child transmission (PMTCT) programs. However, for country wide rollout, a performance evaluation of the South Sudan National HIV test algorithm with a consideration of the SD BIOLINE HIV/syphilis Duo RDT as a first test is recommended.

## Supporting information

S1 File Study dataset. (XLSX)

## References

[1] Wijesooriya NS, Rochat RW, Kamb ML, Turlapati P, Temmerman M, Broutet N, Newman LM: Global burden of maternal and congenital syphilis in 2008 and 2012: a health systems modelling study. *The Lancet Global health* 2016, 4(8):e525–533. 10.1016/S2214-109X(16)30135-8 27443780 PMC6759483

[2] Global HIV/AIDS response: epidemic update and health sector progress towards universal access: progress report 2011. | UNESCO HIV and Health Education Clearinghouse [http://hivhealthclearinghouse.unesco.org/library/documents/global-hivaids-response-epidemic-update-and-health-sector-progress-towards]

[3] World Health Organization: The sexually transmitted diagnostics initiative (SDI): special programme for research and training in tropical diseases (TDR). In. Geneva; 2003: 1–28.

[4] Zetola NM, Klausner JD: Syphilis and HIV infection: an update. *Clinical infectious diseases*: *an official publication of the Infectious Diseases Society of America* 2007, 44(9):1222–1228. 17407043 10.1086/513427

[5] Sellati TJ, Wilkinson DA, Sheffield JS, Koup RA, Radolf JD, Norgard MV: Virulent Treponema pallidum, lipoprotein, and synthetic lipopeptides induce CCR5 on human monocytes and enhance their susceptibility to infection by human immunodeficiency virus type 1. *The Journal of infectious diseases* 2000, 181(1):283–293. 10.1086/315209 10608777

[6] Global guidance on criteria and processes for validation: elimination of mother-to-child transmission (EMTCT) of HIV and syphilis. [http://www.who.int/reproductivehealth/publications/rtis/9789241505888/en/]

[7] Schackman BR, Neukermans CP, Fontain SN, Nolte C, Joseph P, Pape JW, Fitzgerald DW: Cost-effectiveness of rapid syphilis screening in prenatal HIV testing programs in Haiti. *PLoS medicine* 2007, 4(5):e183 10.1371/journal.pmed.0040183 17535105 PMC1880854

[8] Bristow CC, Leon SR, Huang E, Brown BJ, Ramos LB, Vargas SK, Flores JA, Caceres CF, Klausner JD: Field evaluation of a dual rapid diagnostic test for HIV infection and syphilis in Lima, Peru. *Sexually transmitted infections* 2016, 92(3):182–185. 10.1136/sextrans-2015-052326 26670914 PMC4875763

[9] South Sudan Antenatal Clinic Sentinel Surveillance for HIV and Syphilis 2012 [http://ghdx.healthdata.org/record/south-sudan-antenatal-clinic-sentinel-surveillance-hiv-and-syphilis-2012]

[10] Omoding D, Katawera V, Siedner M, Boum Y II: Evaluation of the SD Bioline HIV/Syphilis Duo assay at a rural health center in Southwestern Uganda. *BMC research notes* 2014, 7:746 10.1186/1756-0500-7-746 25339379 PMC4221666

[11] World Health Organisation: WHO prequalification of in vitro diagnostics programmes public report. Product: SD Bioline HIV/syphilis Duo. In.; Oct 2015: 1–10.

[12] Feinstein AR, Cicchetti DV: High agreement but low kappa: I. The problems of two paradoxes. *Journal of clinical epidemiology* 1990, 43(6):543–549. 2348207 10.1016/0895-4356(90)90158-l

[13] Cicchetti DV, Feinstein AR: High agreement but low kappa: II. Resolving the paradoxes. *Journal of clinical epidemiology* 1990, 43(6):551–558. 2189948 10.1016/0895-4356(90)90159-m

[14] Bristow CC, Adu-Sarkodie Y, Ondondo RO, Bukusi EA, Dagnra CA, Oo KY, Pe EH, Khamsay C, Houng le T, Campuzano RV et al: Multisite Laboratory Evaluation of a Dual Human Immunodeficiency Virus (HIV)/Syphilis Point-of-Care Rapid Test for Simultaneous Detection of HIV and Syphilis Infection. *Open forum infectious diseases* 2014, 1(1):ofu015 10.1093/ofid/ofu015 25734088 PMC4324189

[15] Shimelis T, Tadesse E: The diagnostic performance evaluation of the SD BIOLINE HIV/syphilis Duo rapid test in southern Ethiopia: a cross-sectional study. *BMJ open* 2015, 5(4):e007371 10.1136/bmjopen-2014-007371 25908677 PMC4410125

[16] Shakya G, Singh DR, Ojha HC, Ojha CR, Mishra SK, Malla K, Chaudhary P, Regmi K: Evaluation of SD Bioline HIV/syphilis Duo rapid test kits in Nepal. *BMC infectious diseases* 2016, 16(1):450 10.1186/s12879-016-1694-9 27566067 PMC5002177

[17] Black V, Williams BG, Maseko V, Radebe F, Rees HV, Lewis DA: Field evaluation of Standard Diagnostics' Bioline HIV/Syphilis Duo test among female sex workers in Johannesburg, South Africa. *Sexually transmitted infections* 2016. 10.1136/sextrans-2015-052474 27154184

[18] Black V, von Mollendorf CE, Moyes JA, Scott LE, Puren A, Stevens WS: Poor sensitivity of field rapid HIV testing: implications for mother-to-child transmission programme. *BJOG*: *an international journal of obstetrics and gynaecology* 2009, 116(13):1805–1808. 19781042 10.1111/j.1471-0528.2009.02357.x

[19] Moodley D, Moodley P, Ndabandaba T, Esterhuizen T: Reliability of HIV rapid tests is user dependent. *South African medical journal = Suid-Afrikaanse tydskrif vir geneeskunde* 2008, 98(9):707–709. 19113051

[20] Consolidated Guidelines on HIV Testing Services: 5Cs: Consent, Confidentiality, Counselling, Correct Results and Connection 2015. Geneva: World Health Organization 2015.; 2015. 26378328

[21] Olugbenga I, Taiwo O: Clinic-based evaluation study of the diagnostic accuracy of a dual rapid test for the screening of HIV and syphilis in pregnant women in Nigeria. 2018, 13(7):e0198698. 10.1371/journal.pone.0198698 PMC6038984 29990336

[22] Bristow CC, Severe L, Pape JW, Javanbakht M, Lee SJ, Comulada WS, Klausner JD: Dual rapid lateral flow immunoassay fingerstick wholeblood testing for syphilis and HIV infections is acceptable and accurate, Port-au-Prince, Haiti. *BMC infectious diseases* 2016, 16:302 10.1186/s12879-016-1574-3 27316352 PMC4912739

[23] Mabey D, Peeling RW, Ballard R, Benzaken AS, Galban E, Changalucha J, Everett D, Balira R, Fitzgerald D, Joseph P et al: Prospective, multi-centre clinic-based evaluation of four rapid diagnostic tests for syphilis. *Sexually transmitted infections* 2006, 82 Suppl 5:v13–16. 17215274 10.1136/sti.2006.022467 PMC2563907

[24] Causer LM, Kaldor JM, Fairley CK, Donovan B, Karapanagiotidis T, Leslie DE, Robertson PW, McNulty AM, Anderson D, Wand H et al: A laboratory-based evaluation of four rapid point-of-care tests for syphilis. *PloS one* 2014, 9(3):e91504 10.1371/journal.pone.0091504 24618681 PMC3950184

[25] Benzaken AS, Sabido M, Galban EG, Pedroza V, Vasquez F, Araujo A, Peeling RW, Mayaud P: Field evaluation of the performance and testing costs of a rapid point-of-care test for syphilis in a red-light district of Manaus, Brazil. *Sexually transmitted infections* 2008, 84(4):297–302. 10.1136/sti.2007.029462 18305119

[26] Mishra S, Naik B, Venugopal B, Kudur P, Washington R, Becker M, Kenneth J, Jayanna K, Ramesh BM, Isac S et al: Syphilis screening among female sex workers in Bangalore, India: comparison of point-of-care testing and traditional serological approaches. *Sexually transmitted infections* 2010, 86(3):193–198. 10.1136/sti.2009.038778 19965800

[27] Herring AJ, Ballard RC, Pope V, Adegbola RA, Changalucha J, Fitzgerald DW, Hook EW 3rd, Kubanova A, Mananwatte S, Pape JW et al: A multi-centre evaluation of nine rapid, point-of-care syphilis tests using archived sera. Sexually transmitted infections 2006, 82 Suppl 5:v7–12. 17118953 10.1136/sti.2006.022707 PMC2563911

[28] Sena AC, White BL, Sparling PF: Novel Treponema pallidum serologic tests: a paradigm shift in syphilis screening for the 21st century. *Clinical infectious diseases*: *an official publication of the Infectious Diseases Society of America* 2010, 51(6):700–708. 20687840 10.1086/655832

[29] Target Product Profile: Combined HIV/Syphilis Test [http://www.idc-dx.org/resources/target-product-profile-combined-hivsyphilis-test]

[30] Peeling RW, Ye H: Diagnostic tools for preventing and managing maternal and congenital syphilis: an overview. *Bulletin of the World Health Organization* 2004, 82(6):439–446. 15356937 PMC2622868

[31] Watson-Jones D, Changalucha J, Gumodoka B, Weiss H, Rusizoka M, Ndeki L, Whitehouse A, Balira R, Todd J, Ngeleja D et al: Syphilis in pregnancy in Tanzania. I. Impact of maternal syphilis on outcome of pregnancy. *The Journal of infectious diseases* 2002, 186(7):940–947. 10.1086/342952 12232834

[32] Zorzi A, Cordioli M: Field evaluation of two point-of-care tests for syphilis among men who have sex with men, Verona, Italy. 2017, 93(S4):S51–s58. 10.1136/sextrans-2016-053065 29223963

